# Spotlighting healthcare frontline workers´ perceptions on artificial intelligence across the globe

**DOI:** 10.1038/s44401-025-00034-3

**Published:** 2025-07-30

**Authors:** Henrique A. Lima, Pedro H. F. S. Trocoli-couto, Marzia Zaman, Débora C. Engelmann, Rosalind Parkes-Ratanshi, Leah Junck, Brenda Hendry, Amelia Taylor, Michelle El Kawak, Nirmal Ravi, Henrique D. P. Santos, Timothy M. Pawlik, Vivian Resende

**Affiliations:** 1 Department of Surgery, Federal University of Minas Gerais Faculty of Medicine, Belo Horizonte, Minas Gerais Brazil; 2 CMED Health Limited, Dhaka, Bangladesh; 3 United International University, Advanced Intelligent Multidisciplinary Systems Lab, Institute of Research, Innovation, Incubation and Commercialization, Dhaka, Bangladesh; 4 Institute of A.I. in Healthcare, Porto Alegre, RS Brazil; 5 Infectious Diseases Institute, Makerere University, Kampala, Uganda; 6 Clinical School, University of Cambridge, Cambridge, UK; 7 Global Center on AI Governance, Washington D.C, USA; 8 University of Dar es Salaam, Dar es Salaam, Tanzania; 9 Kuyesera AI Lab, Malawi University of Business and Applied Sciences, Thyolo, Malawi; 10 Humanitarian Engineering Initiative, Faculty of Health Sciences and Maroun Semaan Faculty of Engineering and Architecture, American University of Beirut, Beirut, Lebanon; 11 eHealth Africa; EHA Clinics, Kano, Nigeria; 12 Department of Surgery, The Ohio State University Wexner Medical Center and James Comprehensive Cancer Center, Columbus, OH USA

**Keywords:** Health care, Developing world

## Abstract

We sought to define healthcare workers’ (HCW) views on the integration of generative artificial intelligence (AI) into healthcare delivery and to explore the associated challenges, opportunities, and ethical considerations in low- and middle-income countries (LMICs). We analysed unified data from selected 2023 Gates Foundation AI Grand Challenges projects using a mixed-methods, cross-sectional survey evaluated by an international panel across eight countries. Perceptions were rated on a simplified three-point Likert scale (sceptical, practical, enthusiastic). Among 191 frontline HCWs who interacted with AI tools, 617 responses were assessed by nine evaluators. Enthusiastic responses accounted for the majority (75.4%), while 21.6% were practical and only 3.0% were sceptical. The overall interclass correlation coefficient of 0.93 (95%CI: 0.91–0.94, with an average rating *k* = 9) indicated excellent inter-rater reliability. While quantitative data underscored a generally positive attitude towards AI, qualitative findings revealed recurring cultural and linguistic barriers and ethical concerns. This is a unique study analysing data from the first applications of generative AI in health in LMICs. these findings offer early insights into generative AI implementation in LMIC healthcare settings and highlights both its transformative potential and the need for careful policy and contextual adaptation.

## Introduction

While artificial intelligence (AI) cannot claim to replicate human intelligence, it has been utilized in systems that interpret human behaviour and language in specific ways^1^. AI applications may help doctors by analyzing massive amounts of data and supporting more accurate and timely decisions^2^. AI impact on healthcare began with the medical decision support systems (MDSS) such as heart disease diagnostic tool first proposed in 1961^3^. With time, AI has evolved from rigid, rule-based systems to dynamic, data-driven models capable of learning and adapting to different requirements. Early AI was limited to fixed tasks, while modern AI can perform diverse functions, from pattern recognition to content creation. Over time, AI applications have expanded to manage health records, diagnosis, treatment planning, monitoring, and robotic surgery. Predictive AI can forecast outcomes, while generative AI creates new content, mimicking human creativity^4^. Driven by the latter, AI tools now include conversational agents (“chatbots”) powered by large language models with natural language processing to simulate human-like conversations, offering services like health education, mental health support, and quick access to medical information^5^. However, limitations such as emotional understanding and diagnostic accuracy remain profound challenges to their safety and usefulness in different areas.

Healthcare workers (HCW) in Low- and Middle-Income Countries (LMICs) often face compounded challenges, including limited resources, poor infrastructure, and understaffing, making it difficult for them to provide quality care^6^. In these contexts, AI could prove especially useful to improve efficiency, diagnoses, and patient outcomes; however, there are many challenges to the integration of AI in low resource settings. Issues include data privacy, security, cost, expertise and biases in AI models, which can lead to misdiagnosis, particularly in contexts where there is limited contextual digital health data such as rural or minority populations^3^. Many AI models also lack clinical validation and standardized protocols, affecting their quality and safety. In particular, the absence of transparency in how these models make decisions underscores the need for rigorous monitoring and validation processes.

The perception and use of AI vary depending on the context of LMICs. A study in Northern Ghana noted that using a computer-assisted system for antenatal and delivery care reduced complications and maternal deaths, but the cost was high. The training of HCWs was estimated to be $1060 per person, with the largest expense being equipment, posing a problem in poor rural areas^3^. In Bangladesh, a lack of education and training were the main concerns of HCW in applying AI, indicating a discrepancy of attitudes, with medical students demonstrating excitement and doctors having more scepticism^7^. A study from Sri Lanka highlighted a range of challenges in using AI in healthcare, such as legal and ethical issues, data privacy, and security concerns^8^. Researchers from Bhutan raised concerns about the “Black Box Phenomenon” in which AI produces outputs or makes decisions without explaining the rationale. The potential implications for healthcare include concerns about privacy and safety. Notably, 40% of interviewed researchers in Bhutan compared the dangers of AI to those of nuclear weapons^9^.

In 2023, with support from the Bill & Melinda Gates Foundation (BMGF), researchers and innovators in several countries designed, developed, and piloted generative AI-driven healthcare tools based on Large Language Models (LLMs) responding to contextual healthcare problems. Many of them sought to provide support to Frontline Healthcare Workers (FLHCWs). Researchers from the Federal University of Minas Gerais (UFMG), Brazil, and Pakistan examined the potential of LLMs like GPT-3.5, GPT-4, and Meditron-70b in answering maternal health questions. Overall, these models proved effective in providing clear, understandable responses. NoHarm.AI in Brazil was designed to assist clinical pharmacists by automating patient discharge summaries and highlighting critical information such as adverse events, symptoms, and procedures. In Bangladesh, Susastho.AI focused on supporting adolescents with sexual and reproductive health (SRH) and mental health (MH) issues by offering secure and reliable information via a chatbot based on LLM tailored to their needs. Meanwhile, in Malawi, IntelSurv, an LLM-powered web app, made disease-related queries easier, even offline, and streamlined data collection for health workers. In Tanzania, GPT-4 was integrated into community radio, delivering culturally sensitive malaria health messages to underserved populations. By tailoring content with local insights, this initiative reached over 36 million people, improving malaria awareness and contributing to disease prevention in rural and remote areas.

To adopt these tools effectively in the relevant settings, all projects assessed user perceptions through surveys or interviews. This secondary analysis may provide valuable insights to better understand the opportunities and barriers tied to these FLHCW perceptions at a time when LLM and AI in health are in their infancy—but are rapidly being deployed across the globe. Providing a quantitative overview of perceptions while also granularly outlining the strengths and weaknesses of specific models and approaches, this paper provides a critical glimpse into the overall underacknowledged views of FLHCWs engaged in early AI projects across a variety of LMICs at a point in time that is shaped by both (generative) AI optimism and profound fears.

## Results

### Quantitative

**Fig. 1:**
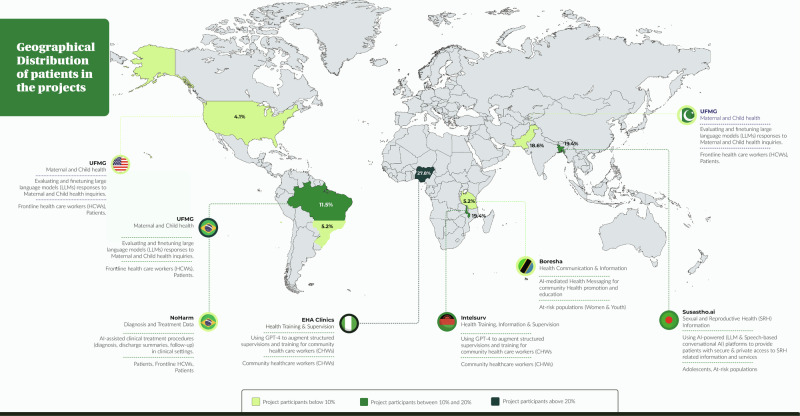
Distribution of participants across the Globe (Created with MapChart).

**Fig. 2:**
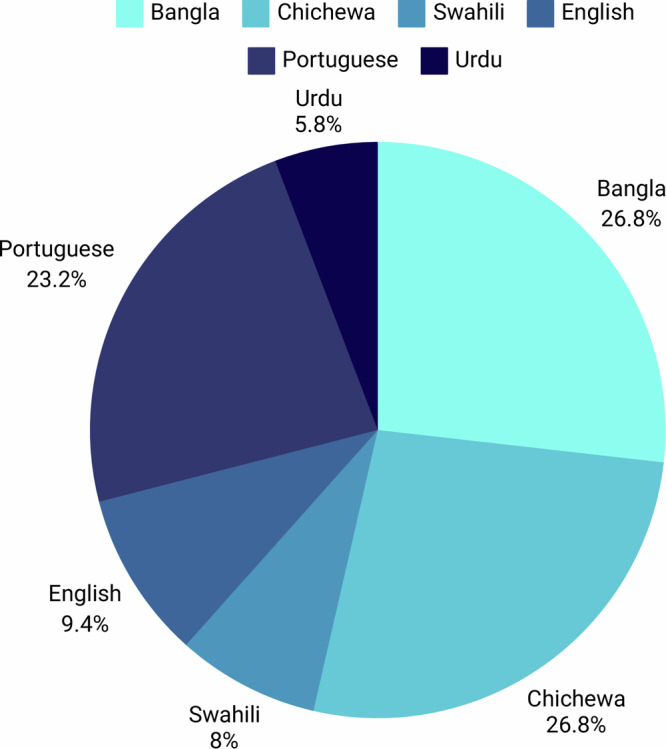
Relative distribution of participant´s languages.

Overall, 191 participants of the primary grantee’s projects generated 617 responses that were extracted and analysed by nine evaluators. The largest number of participants were originally from EHA Clinics project [EHA Clinics 53 (27.8%), followed by UFMG 43 (22.5%), Susastho.ai 37 (19.4%), Intelsurv 37 (19.4%), Boresha 11 (5.8%), NoHarm 10 (5.2%)] (Fig. 1). Just over one-half of the participants was from Africa (101, 52.9%). Regarding the language of participants from which data were derived, there was a relatively even distribution between Bangla, Chichewa and Portuguese [37 (19.4%), 37 (19.4%) and 32 (16.8%), respectively], followed by followed by English, Swahili and Urdu [13 (6.8%), 11 (5.8%) and 8 (4.2%), respectively] (Fig. 1 and 2). The most common source of data was healthcare assistance projects (96 participant responses, 50.3%), followed by healthcare education (85, 44.5%). Only ten (5.2%) participants evaluated the use of AI in healthcare processes (i.e., automated discharge summaries).

Regarding the analysis performed by the nine evaluators, based on average ratings, the two-way fixed-effects consistency model demonstrated an overall ICC of 0.93 (95% CI: 0.91–0.94, with an average rating k = 9), indicating excellent inter-rater reliability.

Overall, most analysed responses came from Susatho.ai project performed in Bangladesh (296, 48.0%), which together with Pakistani responses from UFMG project (30, 4.9%), made Asia as the primary source of analysed responses (326, 52.8%). Most responses were in the context of healthcare education (403, 65.3%), followed by healthcare assistance (194, 31.4%). Notably, responses heavily leaned towards a more positive view, with 432 (75.4%) responses categorised as enthusiastic, 124 (21.6%) as taking a practical view, and only 17 (3.0%) as sceptical. In fact, in three projects (NoHarm, Intelsurv and EHA Clinics) no response was categorised as sceptical. This tendency persisted across studies, countries, regions, languages and social contexts, with a few noticeable exceptions. Although perceptions were largely enthusiastic regarding the use of AI, in NoHarm’s project, which focused on healthcare processes, most perceptions were considered practical [Perception; Practical, 11 (8.9%) vs. Enthusiast, 9 (2.1%), *p* = 0.000*]. Similarly, perceptions regarding responses generated in Urdu (Pakistan) were also mostly practical [Perception; Practical, 7 (5.6%) vs. Enthusiast, 6 (1.4%), *p* = 0.000*] (Table 1).

**Table 1:** Responses from grantee projects by Healthcare Worker Perceptions

Variables	Total *n* = 617 (100%)	Healthcare Worker Perceptions**			*p*-value
		Sceptic *n* = 17 (3.0%)	Practical *n* = 124 (21.6%)	Enthusiast *n* = 432 (75.4%)	
Study					0.000*
UFMG	141 (22.9%)	1 (0.9%)	30 (27.5%)	78 (71.6%)	
NoHarm	20 (3.2%)	0 (0.0%)	11 (55.0%)	9 (45.0%)	
Susastho.ai	296 (48.0%)	14 (4.9%)	67 (23.4%)	205 (71.7%)	
Intelsurv	74 (12.0%)	0 (0.0%)	8 (11.0%)	65 (89.0%)	
Boresha	33 (5.3%)	2 (6.3%)	5 (15.6%)	25 (78.1%)	
EHA Clinics	53 (8.6%)	0 (0.0%)	3 (5.7%)	50 (94.3%)	
Country					0.000*
Brazil	86 (13.9%)	1 (1.2%)	23 (28.4%)	57 (70.4%)	
Pakistan	30 (4.9%)	0 (0.0%)	7 (53.8%)	6 (46.2%)	
USA	45 (7.3%)	0 (0.0%)	11 (31.4%)	24 (68.6%)	
Malawi	74 (12.0%)	0 (0.0%)	8 (11.0%)	65 (89.0%)	
Bangladesh	296 (48.0%)	14 (4.9%)	67 (23.4%)	205 (71.7%)	
Tanzania	33 (5.3%)	2 (6.3%)	5 (15.6%)	25 (78.1%)	
Nigeria	53 (8.6%)	0 (0.0%)	3 (5.7%)	50 (94.3%)	
Region					0.000*
South America	86 (13.9%)	1 (1.2%)	23 (28.4%)	57 (70.4%)	
North America	45 (7.3%)	0 (0.0%)	11 (31.4%)	24 (68.6%)	
Asia	326 (52.8%)	14 (4.7%)	74 (24.7%)	211 (70.6%)	
Africa	160 (25.9%)	2 (1.3%)	16 (10.1%)	140 (88.6%)	
Language					0.001*
English	98 (15.9%)	0 (0.0%)	14 (15.9%)	74 (84.1%)	
Portuguese	86 (13.9%)	1 (1.2%)	23 (28.4%)	57 (70.4%)	
Urdu	30 (4.9%)	0 (0.0%)	7 (53.8%)	6 (46.2%)	
Bangla	296 (48.0%)	14 (4.9%)	67 (23.4%)	205 (71.7%)	
Chichewa	74 (12.0%)	0 (0.0%)	8 (11.0%)	65 (89.0%)	
Swahili	33 (5.3%)	2 (6.3%)	5 (15.6%)	25 (78.1%)	
Context					0.001*
Healthcare Assistance	194 (31.4%)	1 (0.6%)	33 (20.4%)	128 (79.0%)	
Processes	20 (3.2%)	0 (0.0%)	11 (55.0%)	9 (45.0%)	
Education	403 (65.3%)	16 (4.1%)	80 (20.5%)	295 (75.4%)	

*IQR* interquartile range. * signify statistical significance (*p* < 0.05). ** Missing values excluded (44 evaluations).

**Fig. 3:**
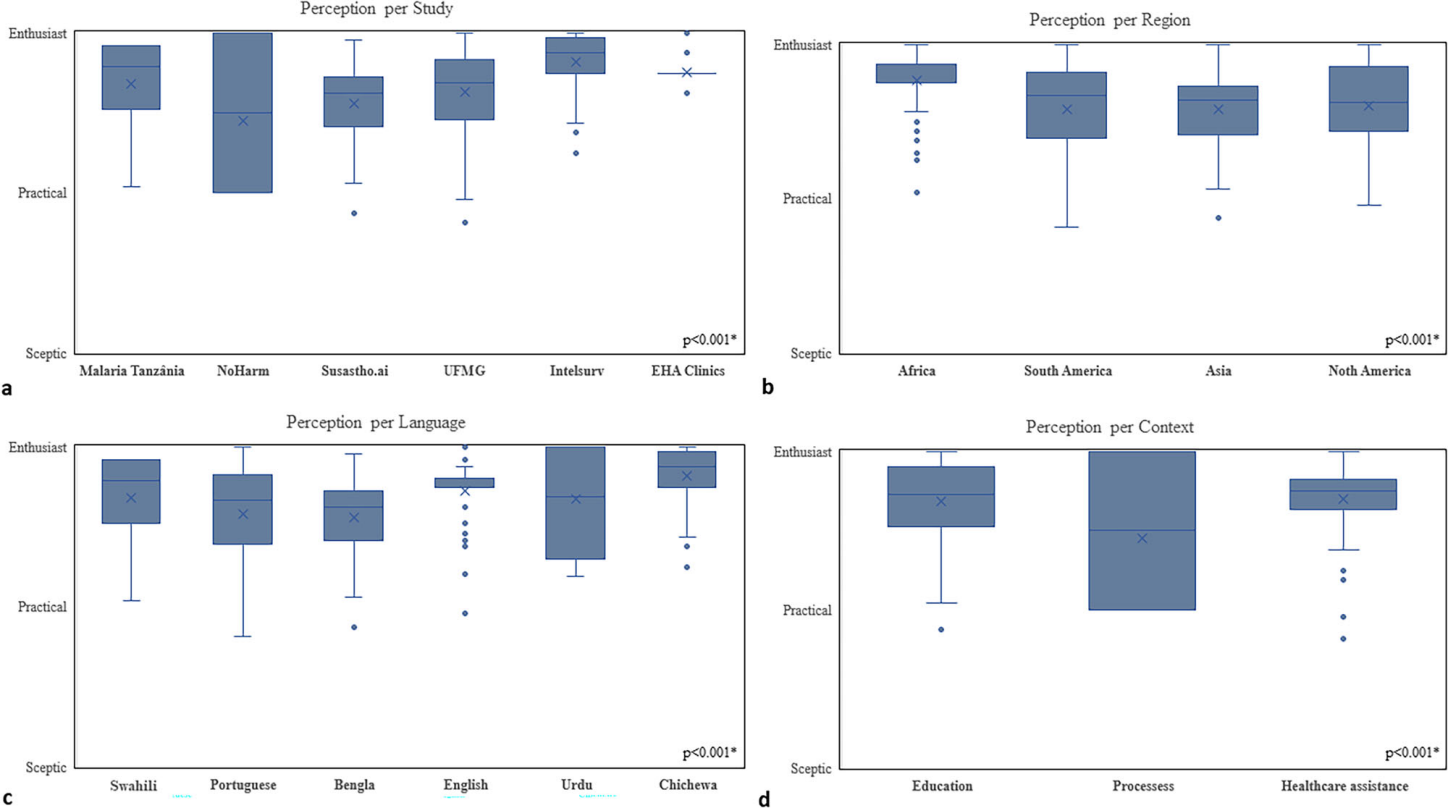
Boxplots depicting averaged overall evaluator perceptions by study. (**a**) region; (**b**) language, (**c**), and context (**d**). Perception classification ranged from sceptic, to practical, to enthusiast; * signify statistical significance (*p* < 0.05).

Considering that each participant’s perception is derived from the combined perception scores assigned to each individual Q&A they analysed, all participants were either practical or enthusiastic regarding the use of AI in their specific setting of application, independent of study, country, region, language, and social context (Fig. 3 and Supplementary Fig. 1). Interestingly, perceptions were most enthusiastic in Africa versus other regions, and it was most heterogeneous among participants who spoke Portuguese or Urdu as their native language (Fig. 3).

### Qualitative

In general, the projects received good feedback regarding general quality of medical information and accuracy of LLMs. However, hallucinations and inaccuracies still occured. There were limitations with some languages such as translation issues in Portuguese and Urdu. Further, complex or more nuanced information such as medical abbreviations were, sometimes, inaccurate. Concerns were raised around context specific and culture specific responses, especially regarding maternal and reproductive health; this was highlighted by poor sentence construction, “politician” type responses or even “scary” language with variable empathy. There was variation in different LLMs, and improvement over the evolution of the projects as new LLMs became available.

### UFMG

Overall, when analyzed by specialists from the United States, LLMs (GPT- 3.5, GPT-4, custom GPT- 3.5, and Meditron-70b) were able to produce good answers effectively to all questions pertaining to maternal health in a fairly clear manner. However, answers often lacked detailed information and sometimes presented outdated medical terms. Moreover, some answers lacked enough description to provide nuanced or site-specific discussion (for instance limited strategies for pain relief during labor). Interestingly, non-trained models outperformed custom LLMs in various scenarios. Overall, LLM responses were characterized by impressively high scores for clarity and quality of content, while readability and poor translation to LIMC languages were identified as key areas of improvement.

### Brazil

When analyzing LLMs (GPT- 3.5, GPT-4, custom GPT- 3.5, and Meditron-70b) performance in responding to questions pretraining to maternal health, specialists pointed out that GPT-3.5 sometimes failed to provide complete information and even omitted potential risks. Examples included not clarifying the possibility of “uterine rupture” in vaginal birth after a caesarean section or not including epidural analgesia as a strategy for pain relief during childbirth. For GPT 4.0, besides mentioning the need for more complete answers, translation errors were often mentioned. Poor sentence construction and mistranslation diminished the authenticity and clarity of information. Answers generated by a custom GPT-3.5 were considered “generic and superficial.” Similarly, Meditron-70b responses were considered generic and incomplete; for example, responses would only address risks and not provide any information about the benefits of certain procedures. Specialists criticized the model’s apparent negative view of labor/delivery, assuming that “there will always be pain that will require some method of relief”, and the failure to address the fact that each woman may react differently according to their tolerance.

### Pakistan

When analyzing LLMs (GPT- 3.5, GPT-4, custom GPT- 3.5, and Meditron-70b) performance in responding to questions pretraining maternal health, answers generated by GPT-3.5 and GPT-4 were generally considered adequate, but sometimes extra information was desired. Custom GPT-3.5 responses lacked sufficient information, overlooked details and omitted important points. In contrast, answers generated by Meditron-70b were often considered very good, containing pertinent and detailed information. However, evaluators noted that Meditron-70b struggled relative to information about the risk of mortality in certain scenarios. Specialists noted that crude information about the risk of death was generated in a “scary way”, without mentioning the available means to mitigate it, and may mislead patients eligible for vaginal birth. Importantly, throughout all LLMs, most criticism regarding responses was related to the reportedly poor Urdu translation and the desire to improve it.

### NOHARM - Brazil

Most healthcare workers noted that traditional summary discharges usually do not provide sufficient information. At the same time, all participants stated that they consider discharges summaries in digital format more convenient. If an automated tool was utilized, all participants stated that they would feel comfortable receiving patient discharge summaries knowing that they were generated by AI and reviewed by a doctor. In fact, 30% reported that they would feel “very comfortable”. Results on the performance of LLMs (GPT- 3.5, GPT-4, MariTalk 1 and 2, and GeminiPro) in automating discharge summaries demonstrated that GPT-4.0 most common mistakes were redundancy (i.e. chronic kidney disease and Chronic Kidney Failure or SS Hemoglobinopathy and Sickle Cell Disease) and incomplete diagnoses [i.e. Type-2 Diabetes (T2D) being referred to as simply Diabetes mellitus; and Dermatitis in the left upper limb (LUL) as only Dermatitis]. Another overall mistake was the presence of non-specific terms when extracting data from patients’ history, such as “Diagnostic hypotheses” instead of the actual diagnoses. Moreover, when asked to work with acronyms, LLMs often misinterpreted them, such as the acronym MCP (“medicamento conforme prescrição”) [MAP (medication according to prescription)], presented by the LLM as “Microangiopatia cerebral proliferativa” [Cerebral proliferative angiopathy]. Although this type of mistake happened in all evaluated models, rates were eight times higher for MariTalk 1 and three times higher for Palm 2. Furthermore, GeminiPro and MariTalk 1 featured hallucinations, suggesting that patients suffered from chronic obstructive pulmonary disease and two of cerebrovascular accident, while no terms in their patients’ history indicated these diagnoses.

### SUSASTHO.AI - Bangladesh

When analyzing LLMs performance as a chatbot in a digital healthcare platform providing secure access to sexual, reproductive, and mental health care for adolescents, most front-line health care workers had a favorable view, describing it as helpful, informative and easy to use and understand. However, some users suggested enhancing the chatbot’s ability to better understand questions and be more comprehensive. Many users felt that the chatbot adequately answered most of their questions with ease and clarity. In contrast, its performance was not consistent and an increased ability to handle complicated or nuanced questions was desired. Most participants saw the chatbot as a helpful and beneficial tool and not a threat. Nevertheless, emphasis on the need for clinically validated and accurate information, as well as minding the occurrence of culturally insensitive information was mentioned. In other words, users felt that with proper management, the chatbot poses no threat and can significantly aid users provided a continuous update and training.

Overall, users noted the Susastho.ai chatbot to be a valuable resource for information on Sexual and Reproductive Health (SRH) and Mental Health (MH). Enhancements in understanding complex questions and expanding the information database are desired. The chatbot holds significant promise for personal use, professional development, rural healthcare support, and breaking down social barriers related to sensitive health topics. Maintaining accurate and validated information is crucial for user trust and the chatbot’s effectiveness.

### INTELSURV - Malawi

When utilizing LLMs (ChatGPT and MedPalm) to develop a tool to streamline the collection, analysis, and use of COVID-19 data, front-line health care workers who navigated the interface encountered no errors or difficulties and managed to ask and receive questions from IntelSurv. The respondents agreed that IntelSurv can improve access to knowledge for their work. They were excited about its ability to answer broad questions about diseases and surveillance other than those typically available in printed materials. Users appreciated the non-restrictive approach that allowed both technical and less technical questions. They remarked positively that IntelSurv was able to correct, understand, and answer questions that contained spelling or grammatical errors. Some areas of improvement noted were regarding response time when answering live questions, the wordiness and length of answers (“the tool answers like a politician”), and the need to add translation to local languages. The top five topics of most interest to participants were cholera, anthrax, how to capture demographic information, the rationale for collecting data, and information about laboratory tests. Cardiovascular diseases and abbreviations also tied in fifth place.

### BORESHA - Tanzania

LLMs have also been tested to automate patient discharge summaries. GPT-4 has proven to be more reliable compared with other models like MariTalk 1 and Palm 2, as it makes fewer errors such as repeating information or simplifying diagnoses. However, LLMs do have flaws. Mistakes like categorizing “Type 2 Diabetes” as just “Diabetes” highlight limitations in providing nuanced output in medical contexts, where these distinctions make a significant difference. In rural areas, chatbots have been integrated with mobile phones or community radio programs and have reached millions with critical malaria prevention messages. These systems adapt responses based on user questions but sometimes struggle with the intricacies of local languages, like Swahili idioms, which slightly reduce their effectiveness. Despite these issues, LLMs have shown to be potentially powerful tools for scaling health education quickly.

Besides improving communication, LLMs were also utilized to help with diagnostics. For instance, predictive tools powered by models like GPT-4 analyzed climate and health data to forecast disease outbreaks, such as malaria surges during rainy seasons. These systems have achieved accuracy rates of up to 85%. However, some healthcare providers worry about over-relying on these systems without proper human validation. AI-powered mental health platforms like locally developed GPT-based systems are offering anonymous, round-the-clock support for issues like anxiety and depression. While these tools help reduce stigma and improve access to mental health resources, they sometimes fail to interpret culturally specific expressions of distress, which underscores the need for human oversight in refining these tools.

Infrastructure and data security challenges pose significant hurdles. A survey of healthcare workers noted that while many are open to using AI tools if data is secure, limited internet access in rural clinics hampers broader adoption. Misunderstandings about AI, like fears it might replace healthcare jobs, further complicate its integration.

## Discussion

The current study provides a nuanced analysis of FLHCWs’ perceptions regarding the integration of artificial intelligence (AI) in healthcare systems across diverse global contexts, particularly in low- and middle-income countries (LMICs). The timing of this research, conducted in conjunction with the rollout of Gates Foundation AI-related grants, offers a unique opportunity to assess early impressions and insights about the implementation of AI in healthcare. These findings not only highlight the potential transformative role of AI but also reflect the challenges associated with deploying novel technologies in resource-constrained environments.

The results demonstrated a broad spectrum of attitudes towards AI adoption, ranging from scepticism to practical acceptance and enthusiasm. These attitudes reflect not only general perceptions of AI, but also specific considerations related to its integration into healthcare delivery systems. For example, “enthusiasts” frequently emphasized the ability of AI tools to enhance efficiency, improve diagnostic accuracy, and streamline workflows, directly impacting their ability to deliver timely and effective patient care. One respondent noted that “AI has the potential to reduce the time spent on routine tasks, allowing us to focus on patient care”. Conversely, “sceptics” highlighted concerns such as mistrust in automated processes and cultural or linguistic mismatches, which could hinder the effective implementation of AI tools. As one participant in South Asia remarked, “the translations are often too literal, and they miss the nuances of our language”. These divergent views underscore the need for localized training, trust-building initiatives, and iterative development of AI tools to meet the specific needs of healthcare workers and ensure seamless integration into healthcare systems.

Considering the well-documented gaps in communication within healthcare settings, the results build on the understanding of the role of culture and language as significant obstacles^10^. Cultural and linguistic barriers emerged as critical challenges, particularly in regions such as South Asia and Sub-Saharan Africa. For instance, AI models often struggled with idiomatic expressions or technical terminologies specific to local healthcare contexts. In Brazil, respondents stressed the need for AI tools to provide more actionable and accurate responses to maternal health queries, while in Malawi, participants praised AI for improving access to real-time knowledge during disease surveillance but highlighted the necessity of offline functionality to address inconsistent internet access. These observations suggest that user feedback is not always categorical but instead varies across different aspects of the technology, such as usability, accuracy, and adaptability to specific contexts. Paying close attention to these distinctions is critical for effective implementation and evaluation.

Ethical dimensions were a recurring theme in participant feedback. Concerns about data privacy, biases in AI algorithms, and the potential for oversimplified or generalized outputs were frequently mentioned. One healthcare worker commented, “AI often gives generic advice that lacks the context-specific insights we need for patient care”. This finding emphasizes the importance of transparency when addressing the trade-offs of these technologies, allowing stakeholders to learn from diverse implementations and avoid potentially significant mistakes. Beyond the ethical concerns identified, broader barriers such as insufficient funding, weak governance frameworks, and the risk of over-reliance on AI systems—potentially leading to diagnostic errors or diminished clinical skills—also warrant attention. Particular caution is needed when deploying AI in sensitive areas like mental health, where human oversight remains indispensable.

Tensions between quantitative and qualitative findings illustrate the shortcomings of quantitative or survey-based approaches, which, by themselves, may capture a general sentiment but neglect legitimate concerns that require a more qualitative and ideally longitudinal approach to address. Moreover, it is important to note that variability in the number of responses per participant may influence specific sub-group quantitative analysis, as individuals with more responses may disproportionately influence quantitative overall perception trends. Nevertheless, an example is respondents working on Urdu and Portuguese LLMs expressing enthusiasm in the quantitative findings, while these were also the precise languages that proved the most challenging in applications. This corresponds with an overall discrepancy between quantitative findings, suggesting a blanket positive view, and qualitative findings, pointing at various context-specific challenges that emerge when employing generative AI tools to support FLHCWs, particularly when catering to different languages. Addressing these issues is even more critical in resource-limited settings, where reliance on technology to supplement health information carries higher stakes.

When interpreting these results, it is important to acknowledge the limitations of this study. The data were derived from an opportunistic analysis of responses routinely collected during ongoing projects, rather than through a prospectively designed study specifically aimed at addressing these questions. As a result, some insights required extrapolation, and certain nuances may have been missed. Therefore, findings should be generalized cautiously, primarily to early-stage AI implementations in similar LMIC settings, acknowledging that they may not capture the full heterogeneity of all LMIC. A further limitation was the absence of detailed demographic data, which precluded analysis of how perceptions might vary by age, gender, education, or professional background. Additionally, while this study offers a broad overview, future research should explore specific AI applications in greater depth to capture more nuanced insights. A future prospective study, specifically designed to examine healthcare workers’ perceptions of AI, could provide a more comprehensive and focused understanding. This is an important research area, as the adoption of technologies in health settings is a complex issue, and understanding HCW perceptions is essential for AI to be deployed in a sensitive and effective manner. While data from our study reflects voluntary contributions from different projects, leading to an uneven distribution, it captures diverse healthcare applications and settings, offering valuable early insights into frontline workers’ perceptions across LMICs. Finally, as the study is cross-sectional, it reflects initial impressions rather than long-term attitudes, which may shift with prolonged use and real-world integration, as AI tools continue to advance and adapt to LMIC healthcare environments.

Nonetheless, the very timing of collecting the feedback that informs this paper and the fact that FLHCWs could express their views concerning a specific project that directly speaks to their everyday realities also makes the findings unique. It also renders the circumstance telling that participants, despite the challenges they identified, recognized the fundamental potential of AI tools to address systemic gaps in healthcare delivery. This suggests a promising outlook for their applicability and usefulness. Applications such as automated discharge summaries and chatbot-based health education, for instance, were seen as promising innovations, while issues such as repetitive errors, mistranslations, and misinterpretation of medical abbreviations were highlighted as areas for improvement.

This study aims to analyse implementation data drawn from rapid response projects in diverse LMIC settings undertaken in the very early days of widespread LLM; whilst there are limitations with this analysis, we believe there also important insights into common themes in healthcare worker opinions across different contexts. This study highlights the dual role of AI as both a catalyst for healthcare innovation and a subject of critical scrutiny. The findings underscore the need for tailored implementation strategies that prioritize inclusivity and context-awareness, alongside continuous engagement with healthcare workers. By addressing cultural and linguistic barriers, ensuring ethical safeguards, and fostering trust-building initiatives, AI can be more effectively integrated into healthcare delivery systems.

Policymakers should focus on strengthening digital infrastructure, particularly in rural areas, to enable broader access to AI tools. Additionally, fostering collaborations among AI developers, healthcare practitioners, and patients will be crucial to ensuring tools are designed with end-users in mind. Efforts should also explore the scalability, cost-effectiveness, and seamless integration of AI tools into existing workflows.

Building on these findings, future research should include longitudinal studies to evaluate the sustained impact of AI adoption on healthcare outcomes, particularly in underserved communities. Incorporating feedback loops to refine AI outputs based on real-world applications will be essential to maintaining trust and relevance.

By addressing the challenges identified and leveraging the insights provided by healthcare workers, stakeholders can advance the development of equitable, efficient, and impactful AI-driven healthcare systems globally.

## Methods

This cross-sectional study adopted a mixed-methods approach, utilizing both quantitative and qualitative evaluation techniques to investigate the perceptions of frontline healthcare workers toward the use of AI tools in LMICs. Nine evaluators assessed the perception of FLHCWs on the use of AI in different settings. This study was approved by the Institutional Review Board of all grantee institutions. All interactions with the LLM were conducted in compliance with OpenAI’s use case policy and The Bill and Melinda Gates Foundation policies^11^.

### Data Selection

We approached all grantees working on health projects in the *2023 Grand Challenges: Catalyzing Equitable Artificial Intelligence (AI) Use to Improve Global Health*. All projects reviewed their routinely collected data and extracted relevant information that could contribute to the larger study dataset. This was determined by the availability of structured, analyzable perception data at the time of data collection. Of note, each project was evaluating different LLMs, therefore, data source differed on various tools. Nevertheless, a set of variables, from each project, containing questions and answers that pertained to healthcare frontline workers´ perceptions on the use of AI was extracted by each team. Of note, despite different contexts of AI application, the respondent population was consistently healthcare workers across all projects. Of 31 health projects, six teams provided appropriate data for this study. Depending on the topic that the project was initially conducted, the projects were categorized by the authors in three different contexts: Healthcare Assistance (UFMG and EHA Clinics), Education (NoHarm) or Processes (Susatho, IntelSurv and Boresha). Table 2 provides details on these projects. These data were combined into one spreadsheet and sent to evaluators as a survey for analysis (Supplementary Table 1**)**.

**Table 2:** Characteristics of the Projects

Name	Type	Aim	Country	End-Users Context	Languages Used
UFMG	Maternal and Child health	Evaluating and finetuning large language models (LLMs) responses to Maternal and Child health inquiries.	Brazil, Pakistan, USA	Frontline health care workers (HCWs), Patients	Portuguese, Urdu, English
NoHarm	Diagnosis and Treatment Data	AI-assisted clinical treatment procedures (diagnosis, discharge summaries, follow-up) in clinical settings.	Brazil	Patients, Frontline HCWs, Patients	Portuguese
Susastho.ai	Sexual and Reproductive Health (SRH) Information	Using AI-powered (LLM & Speech-based conversational AI) platforms to provide patients with secure & private access to SRH related information and services	Bangladesh	Adolescents, At-risk populations	Bangla
Intelsurv	Health Training, Information & Supervision	Using GPT-4 to augment structured supervisions and training for community health care workers (CHWs)	Malawi	Community healthcare workers (CHWs)	Chichewa
EHA Clinics	Health Training & Supervision		Nigeria		English
Boresha	Health Communication & Information	AI-mediated Health Messaging for community Health promotion and education	Tanzania	At-risk populations (Women & Youth)	Swahili

*UFMG* Universidade Federal de Minas Gerais, *USA* United States of America, *GPT* Generative Pre-Trained Transformer.

### Evaluation

The evaluation was conducted by an international panel of nine evaluators from Brazil, Malawi, Lebanon, Tanzania and Nigeria identified by the nine grantee teams on availability and expertise. Each of them was presented with a standardized spreadsheet that included all sets of questions & answers from each project that addressed the main topic of this paper, resulting in a total of 617 responses per evaluator. Responses were assessed according to a three-point simplified Likert scale: *sceptical*, indicating the least positive attitude toward AI use in their setting; *practical*, reflecting a neutral stance without strong opinions on AI’s usefulness; and *enthusiastic*, representing the most positive attitude toward AI adoption in their setting. Missing data were designated as N/A (non-available)^12^.

To ensure the objectivity and reliability of the evaluation process, each evaluator independently assessed the responses. To safeguard against potential bias from peer influence, evaluators were blinded to the assessments made by their colleagues. All data was in English or had been translated into English by the project team before submission.

Qualitative analysis was performed by reviewing the written feedback provided in each grantees project’s final report. This review was performed by the lead of each six teams included in this project as well as the authors. These reports provided granular data to identify areas of improvement, such as incorrect, incomplete or inadequate content, as well as notable strengths in the use of AI by healthcare workers for each project.

### Statistical Analysis

The non-parametric Kruskal-Wallis test was utilized to compare scores between different answers due to the non-normal distribution of the data evidenced by the Shapiro-Wilk test. Categorical variables were presented as numbers and percentages and compared with the chi-square test, or Fisher exact test, to evaluate the hypothesis of independence. Continuous variables were presented as medians with interquartile range (IQR) and compared using Kruskal-Wallis test; and descriptive statistics included means and standard deviation (SD). Given the skewed, ordinal nature of the data, intraclass correlation coefficient (ICC) was employed to evaluate inter-rater reliability. Specifically, we used an average rating, fixed-effects consistency ICC model, focusing on the correlation of ratings among evaluators rather than their absolute agreement^13,14^. All tests were 2-sided, *p* < 0.05 was considered statistically significant, and post-hoc adjustments using the Bonferroni correction were applied to adjust for multiple comparisons^15^. All statistical analyses were performed using SPSS software version 28.0 (IBM Corporation, Armonk, NY).

## Supplementary information

Supplementary Material

## Data Availability

Data is provided within the manuscript or supplementary information files.
