# Supplementary material for: Spotlighting healthcare frontline workers´ perceptions on artificial intelligence across the globe
Source: Npj Health Syst. 2025 Jul 30;2:28. doi: 10.1038/s44401-025-00034-3 (PMC12310525; doi:10.1038/s44401-025-00034-3)
Supplement: Supplementary file 1 — Supplementary Material [file 44401_2025_34_MOESM1_ESM.pdf]

Supplementary Material

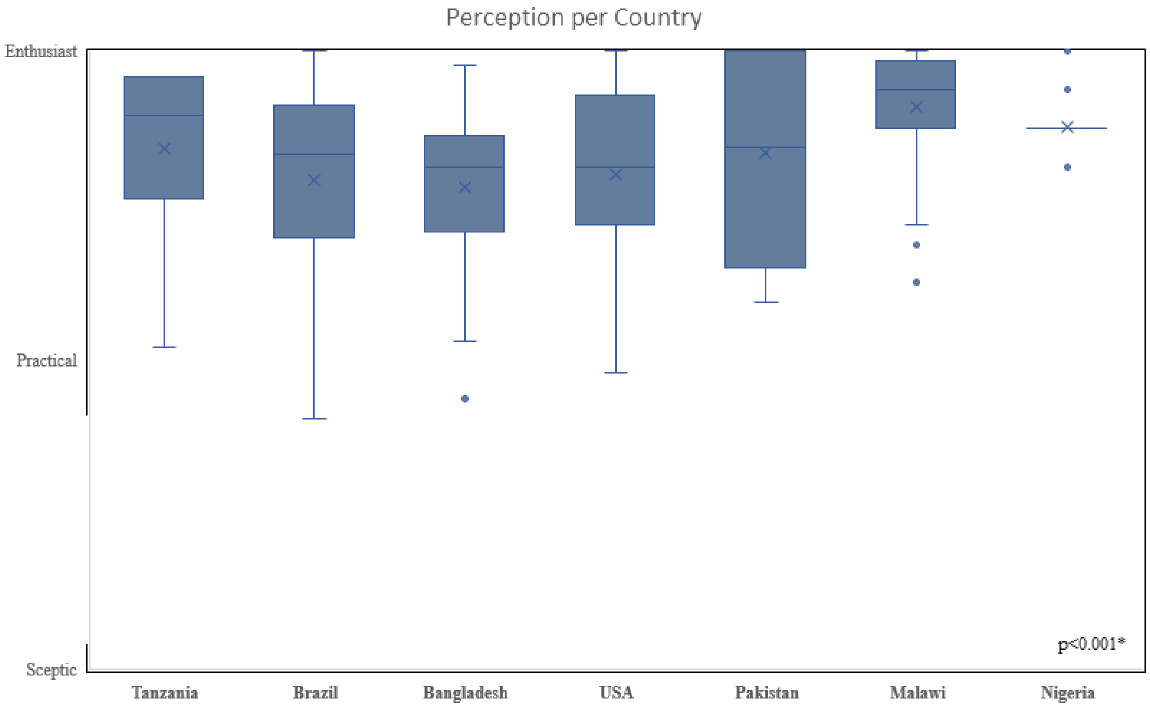

Supplementary Figure 1. Boxplot depicting averaged overall evaluator perceptions by country. Perception classification ranged from sceptic, to practical, to enthusiast; * signify statistical significance ($p < 0.05$).

Supplementary Table 1. Questions and Answers overview

Study	Questions & Answers	
Boresha	Question 1	
		Confidence
	Answer	
		5
		5
		5
		4
		5
		4
		4
		Yes
		4
		5
		5
	Question 2	
		About ChatGPT
	Answer	
		Increased program efficiency
		Ease of information access
		Increased knowledge
		Increased ability to obtain and convey information effectively to the community
		How to open and close a session
		Getting preliminary information during program preparation
		Helps prepare effective messages and programs for the community
		Use in health programs
		Helps in information collection and obtaining proper arrangements
		Increases understanding
		Increased work efficiency
NoHarm	Question 1	
		How would you feel if you knew that Artificial Intelligence (AI) was used to create a patient's discharge summary and reviewed by the doctor?
	Answer	
		Very comfortable - I have full trust in AI and believe it can improve the accuracy and efficiency of the discharge summaries.
		Comfortable - I have some trust in AI, but I still believe that human review is essential.
		Comfortable - I have some trust in AI, but I still believe that human review is essential.
		Very comfortable - I have full trust in AI and believe it can improve the accuracy and efficiency of the discharge summaries.
		Comfortable - I have some trust in AI, but I still believe that human review is essential.
		Very comfortable - I have full trust in AI and believe it can improve the accuracy and efficiency of the discharge summaries.
		Comfortable - I have some trust in AI, but I still believe that human review is essential.
		Comfortable - I have some trust in AI, but I still believe that human review is essential.
		Comfortable - I have some trust in AI, but I still believe that human review is essential.
		Comfortable - I have some trust in AI, but I still believe that human review is essential.
	Question 2	

		Based on what has been presented (here you would show the system), do you think our new system would meet your needs for receiving discharge summaries?
	Answer	
		I completely agree.<br>I partially agree.<br>I completely agree.<br>I completely agree.<br>I completely agree.<br>I completely agree.<br>I completely agree.<br>I partially agree.<br>I partially agree.<br>I partially agree.
Susastho.ai	Question 1	
		What is your perception about the chatbot?
	Answer	
		Initiatives to develop chatbots and more should be developed<br>User friendly.<br>I find this very helpful.<br>the chatbot is helpful.<br>Yes. It is very responsive and informative.<br>It is nice. but it needs a little improvement, such as it would be better to understand the question<br>It is fantastic.<br>Helpful<br>Overall good<br>It is good<br>It provided good answers to the majority of my questions<br>the chatbot is useful , authentic answer can be found.<br>good<br>The chatbot has seemed very easy and fluent to me. I have received answers to all of my questions very easily. It's even better that the chatbot is in Bengali. I hope we will get a lot of benefit from it.<br>The chatbot is rich in information and helpful. However, more information needs to be added<br>it is helpful<br>it is very good and easy to understand.<br>It is good but need more improvement.<br>It is excellent<br>Good<br>It Is good<br>It is useful<br>It is very good<br>It is good<br>It is good<br>It is moderately good.<br>It is comparatively good<br>It is very good<br>It is comparatively good
	Question 2	

Did the response from the chatbot answer your question? If not, please let us know how closely it met your needs.

Answer

Sometimes it works, sometimes it doesn't.
It was able to give answer all the questions except for a few
Yes. It was able to give most of the answers.
Yes
Yes
Yes. It was able to give most of the answers
It was able to give 90% answers.
It's better than before
It was able to give the correct answers to the questions
Yes.
Yes, except for a few complicated ones
No, it couldn't answer all of them; it answered incorrect for about 20%
It provided some answers but couldn't provide others
The chatbot was able to give answer all my questions. I found the answers very easy to understand. I learned many important topics related to sexual and reproductive health as well as mental health.
I received answers to seventy to eighty percent of the questions
Yes, it was able to.
Yes,it can managed to answer most of the questions except some tricky questions
it was able to give most of answers.
it was able to give a little amount answer.
it was able to give a moderate amount answer.
Yes
It provided some answers but couldn't provide others
It was able to give 90% answers.
Yes, it was able to.
Yes, it was able to give almost all questions.
It was able to give 60% answers.
it was able to give most of answers.
Yes, it was able to give answers. I understood it very well.
It provided some answers but couldn't provide others

Question 3

How can the chatbot contribute to your personal and professional life?

Answer

It can help me to my work place by guiding adolescents regarding sexual, reproductive and mental health.
It helps to get accurate informations & act as medium to get early consultations.
It could provide solutions to various types of mental health issues
The chatbot can help knowing unknown information in personal life and provide quick solution to mental health information and advice. In professional life, it can gather basic information and offer quick answers and advice, improving work efficiency
It can greatly enhance knowledge about sexual reproduction and mental health, which is very important for personal life. In our professional lives, we often face significant stress and pressure. The chatbot can provide valuable information to help relieve this stress and improve our mental health.
In my personal life, I suggest that it is very important, but I have never used it in my professional life.
it is important.
I can learn a lot of information. I can learn many confidential information in my personal life. It helps me provide information to everyone in my professional life .
The chatbot can help by giving correct and balanced answers to the questions.

It will play a special role in Health related matters .
It can provide helpful counseling on topics that can be rather difficult to share with other people.
There is a lot of information available into Google searches, and in that case, a chatbot can gather scattered information together which making it easy to access the data.
It will help me in providing health information.
The chatbot can play a significant role in both personal and professional life, providing us with many unknown insights. Through this, we can become much more aware in our personal and work lives.
It will be helpful in providing treatment. I think it will be useful to know, inform and satisfy people's curiosity.
I can easily get answers to my various health related questions which can't be asked directly to the doctor for fear of embarrassment.
It allows me to ask questions about sexual reproduction and mental health in a safe environment, which may be a little uncomfortable to tell someone else.
Yes
All the answers about sexual health are easily available,which can help prevent many issues,
It was perfect
It can help improving mental health and raising awareness about sexual and reproductive health.
yes
There will be many roles
By solving various problems
There will be many roles
It can help by providing accurate information about diseases and giving names and addresses of specialist doctors.
I have no idea about this.
It's a great initiative and can play a significant role. For example, I've been able to find answers to many questions that I didn't know before.
In many ways

Question 4

How can it serve as a helpful information resource for health workers?

Answer

Diseases or health issues information: Health workers can quickly access accurate information on SRH (Sexual and Reproductive Health) and MH (Mental Health). This information can assist in providing proper advice to patients.
It can help by giving correct answers.
By providing various types of information.
A medical chatbot can act as a helpful resource for healthcare professionals by providing quick medical information, following guidelines, offering new updates, and facilitating training.
It will serve as a valuable reference for health workers while maintaining accuracy in the medical field.
I believe that health workers can enhance their knowledge from this chatbot and learn or gain insights from the information provided, which can assist in giving health advice or delivering healthcare services.
Health workers will learn a lot of unknown information.
Healthcare professionals will be able to monitor SRH and MH and easily get answers to any related questions.
This platform helps me getting a lot of information about health and aids in raising awareness about their future.
By providing correct and relevant answers to related questions.
They will be able to learn new health-related information, which will benefit them.
They can use it to ask about their concerns or to gain knowledge on topics related to sexual reproduction and mental health.
Since the information is authentic, it can be confidently shared with the general public without hesitation.
It can serve as a collaborator in providing clinical information.
With the help of chatbots, health workers can learn more information more easily and in less time and can better understand patients about sexual and reproductive health and mental health.
I think it will be useful to know, inform and satisfy people's curiosity.
Through this, healthcare professionals will be able to get ideas about the primary treatment of various diseases
Healthcare professionals can use this to expand their knowledge and, otherwise, understand patients' concerns that they might feel embarrassed to share.
by giving health advice

To know all things
good
Provides information and knowledge on appropriate topics
From here, information about various diseases can be collected, which will serve as a helpful resource for healthcare professionals.

By suggesting the treatment of the disease

I think it needs to be updated further, but it can still serve as a decent resource for information.
comparatively can give answers.
This provides good helpful information for health workers which will be very useful in future.
Very good as a helper

Question 5

If patients use this to ask questions related to SRH (Sexual and Reproductive Health) and MH (Mental Health), how do you think it would be helpful in providing advice?

Answer

"Increase in mental health awareness: The chatbot can facilitate open discussions about mental health and raise awareness among patients about the early signs of mental disorders. This will help patients feel more comfortable discussing their issues."
It will be very helpful. Patients will receive guidance on their questions related to sexual and reproductive health as well as mental health.
Questions from patients provide insights into their real problems and their mental and physical health status, which helps the doctor or consultant to give the right advice.
If a patient mentions specific symptoms or issues, the chatbot can suggest seeing a doctor or recommend some preliminary tests, which can aid in identifying health problems. In the chatbot, patients can discuss health issues and raise health awareness, which can be effective in counseling and treatment.
If a victim needs counseling on sexual reproduction and mental health, they can easily get their information using this chatbot.They just need to ask about the issues they are facing regarding their sexual reproductive and mental health.
The fear that prevents people from seeking information from others due to embarrassment will no longer be an issue now.
by giving answer about SRH & MH.
This platform provides a lot of information. Helping everyone to be aware with a lot of information. I personally got a lot of information
By providing correct and relevant answers to related questions.
They will get answers to personal questions. Which will help them.
It can provide advice & counseling both to make them feel at ease & help them making the right choice
Common people have many misconceptions about sexual health, they see a lot of information on the internet, in that case I think chatbot will benefit them by providing correct information.
People have various misconceptions about SRH and MH and get wrong information from various online portals. Susastho AI can play a role as a clinically validated platform to avoid these misinformation.
The chatbot answers all the questions very easily and fluently thereby facilitating the understanding of the patients.
It can answer most of the questions and help reduce people's misconceptions.
patients will be able to get correct answer in very less time
Patients can use it to easily get answers to their reproductive and mental health questions
will be good
It would be better if the answers were a little easy.
will be good
It will give us an idea about mental health and sexual reproductive health which is a very important topic and we can use it to know the right information.
It will help by suggesting how to prevent it in the first place.
It will be very good for people and the society will also become very beautiful
with help
I don't know
It cannot understand all types of questions because not all patients know the exact names of their conditions. If patients ask questions in their own words, it may not be able to provide answers.

Actually, I never thought that way, so I don't think anything at this moment
I think it will be very helpful.
dont know

Question 6

To know about SRH (Sexual and Reproductive Health) and MH (Mental Health), How can Susastho AI chatbot play a role in rural areas?

Answer

Chatbots for rural communities can provide important information on sexual health, reproductive health, and mental health in simple language, such as sexually transmitted infections, safe pregnancy, family planning, and symptoms and solutions for mental disorders.
Through this, teenagers in rural areas will get proper information about their mental and sexual and reproductive health.
In rural areas people do not get proper health awareness due to lack of health information and misconceptions. The Susastho.AI chatbot is available 24/7. Getting the right information quickly and easily is possible using Susastho AIchatbots.
In rural areas, it is often difficult to discuss sexual and reproductive health and mental health openly, as people may feel embarrassed to talk about these topics and are hesitant to express their concerns. The SuSastho.AI chatbot provides personal and confidential advice, encouraging individuals in rural areas to seek information and ask questions comfortably.
The chatbot provides information and can help raise awareness about sexual and reproductive health and mental health in rural areas.
It will play a very important role in the rural areas but the challenge is to know whether the people at the rural level have smart phones.
Most of the people in rural areas are very ignorant about these things and are afraid to ask anyone about these things.so by using this they can use this to learn on their own.
Everyone should be encouraged to use this chatbot.It should help them by providing confidential information on various topics.
There are many superstitions related to rural areas which this chatbot can easily remove.
They will know about health very easily.
Village people will be benefited greatly as they are not very aware of these topics & as some of them are considered taboo. Also they can easily access the device as the chatbot is in Bangla & also have Text to Speech and ASR features.
There is shortage of health care and staff in rural areas. They get wrong information from local hawkers, Kaviraj. If they are informed about this bot they will benefit from correct information.
People in rural areas are the most backward in terms of information. In this case it can act as a validated site.
People in rural areas will get answers to all questions very easily and in Bengali, so it will be very beneficial for them to use it.
It will help to satisfy the curiosity of rural people and reduce misconceptions.
In rural areas where proper treatment is not readily available, they can get proper advice using this chat bot
It will play a very important role in rural areas as people in those areas are not very aware of these issues and hesitate to talk about these issues.
moderately good
Know all the information about healthy sexual and reproductive health
good
It will play a special role because if people who don't know about sexual and major health and mental health can use it, I think they will get a clear idea from it.
Will play an important role. In rural areas it is not always possible to go to the doctor to know how to cure the disease in the first place, then with the help of chatbot it can be solved at home in a short time. So it will play an important role in rural areas. .
Through them both reproductive health and mental health will be much better at rural level
Finding and solving their problems through the information
I have no idea
As people in rural areas are not much educated, they need to be updated more about regional languages.
It will play a good role in this case because many people don't know the general things.
Just as this chatbot has helped us by answering our questions, I hope it continues to provide answers to any unknown questions in the future.

Question 7

How do you think your family members, colleagues, and friends could benefit from using the chatbot?

Answer

Family: If someone in my family has concerns about sexual or mental health, they can find solutions confidentially through the SuSastho AI chatbot. This can be a comfortable option for many who feel hesitant to speak directly to someone.
By providing the right solution and the right functionality.

Family, colleagues and friends can easily access health information, such as common diseases, mental health, sexual and reproductive health. Using chatbots can get accurate information and tips about stress, anxiety, depression, etc., which will help them stay mentally healthy.
One can obtain health-related information and basic advice from the chatbot. The chatbot can help address sexual and reproductive health and mental health issues, promote health awareness, and provide valuable advice on mental health relationships.
I think my family members, friends, colleagues will benefit from using this chatbot because they don't need to get information about sexual reproduction and mental health from any health counselor or anyone else they can get information and increase their knowledge from chatbot.
Can get all the basic health information.
Everyone will be able to find answers to their unknown questions on their own, without having to ask anyone else.
They can be physically and mentally aware.
By providing correct and relevant answers to related questions.
Everyone will benefit from having their own questions answered.
They will be able to share their concerns & questions freely without the though of being judged.
Benefit from accurate information about sexual and mental health
SRH & MH is a sensitive issue. Especially the youth tries to know more about this. So Susastho AI can play a helpful role in getting the right information.
By using the chatbot, your family members, colleagues, and friends will be able to know about everything in less time, easily, and be more aware than before.
They will receive primary information about any type of sexual, reproductive, and mental health issues, along with knowledge about guidelines on what actions to take.
It was like the right advice at your fingertips
By using it, I or my family and friends can easily find any information related to sexual reproduction and mental health at home and be safe from common misconceptions about these topics in the society.
good

good
One will learn about mental health and sexual reproductive health
Being aware of the disease and its prevention in a short period of time at home can be beneficial in treating the disease early.
There are some problems that cannot be told to parents and brothers. You can solve your own problems through AI

I have no idea
Its quite ok for basic concepts.
As I am a medical student myself, I am enough for my family inshallah.. no need for chat
The way the chatbot has given us answers to many questions that we didn't know so that we can get the answers to these questions later on should be taken care of. Remember to keep this chatbot running.

Question 8

Can such chatbots cause a threat to people? If so, how?

Answer

No
No
No
No
No
No
No
Everyone will depend on this platform. Then they will not discuss among themselves.
no, Such chat bots are life saving.
no
I don't think so, on the contrary I believe it to be quite helpful for people across age or gender.
In the age of information technology, getting accurate information is not a threat but a blessing.

		This is a physical and mental health information site. Which can help in providing correct information. If the information is properly validated then there is no question of threats.<br>I don't think chatbots are a threat to humans.<br>Sexual, reproductive and mental health are sensitive issues. Personal, family and social problems may arise if information is provided that is wrong or contrary to society's values.<br>I think it is convenient for everyone<br>Not at all, rather it will act as a great friend for humans<br>no<br>no<br>no<br>no<br>no<br>no<br>no<br>No. It is very helpful for people<br>Yes, because he can't give correct information due to not updating much. As a result of which people may be harmed and may even die.<br>I don't think such chatbots are a threat to humans.. because these chatbots are doing what they can solve or do. And the one who can't, informs about that and advises to go to the doctor..<br>No, not threatening.
UFMG	Question 1	Considering that empathy is the ability to put oneself in someone else's shoes, perceive their emotions, and understand their perspectives, do you consider the generated answers you evaluated are empathetic?
	Answer	Yes<br>Yes<br>Neutral<br>Neutral<br>Neutral<br>Yes<br>Neutral<br>Yes<br>Yes<br>Yes<br>No<br>Neutral<br>Yes<br>Yes<br>Neutral<br>Yes<br>Yes<br>Neutral<br>Yes<br>NA<br>Yes<br>Neutral<br>Neutral<br>Yes<br>NA<br>NA

Yes
Yes
Neutral
Yes
Yes
Yes
Yes
Neutral
Yes
NA
Yes
Neutral
Yes
Yes
Yes
Yes
Yes
Yes
Neutral
Yes
Yes

Question 2

Based on the experience gathered in evaluating the generated answers, what aspects would you highlight as positive regarding AI models as a source of high-quality medical information?

Answer

The ability to generate accurate well written answers to common questions can be a time saving measure for clinical practices.
Assuming reliable sources for input (NIH, National Societies and not every internet data source), it's good synthesis of the information. I particularly liked the emphasis on reviewing individual health situations with the doctor/CNM.
Ability to summarize information in short easy-to-read bits
ability to quickly answer common questions with standard of care information using appropriate non technical language.
No shaming noted in tone.
Patients have immediate access to info
Information is generally good
I didn't find anything that was inaccurate.
I consider it positive the ability to cover frequently asked questions in a practical way.
Wide access to important information.
Speed of access.
Accessibility to potential quality information.
NA
Yes
Correct information in all responses.
NA
NA
NA
NA
NA
NA

AI is able to generate answers that are written at a level that is clear and easy to understand, and also accurate. Also identifying that the response was generated in this way is also important
Need to balance brevity with providing enough information. Need to acknowledge variations from the norm and be affirming.
focus on shared decision making in some of them
NA
NA
It is a great initial source of important information.
Ease of interaction with the software, in natural language.
NA
They are capable of searching and synthesizing large amounts of information.
It seems to be an interesting option for accessing knowledge, as long as it is used with seriousness and care.
The models can be quite comprehensive and contain scientifically reliable information.
NA
NA
NA
NA
NA
The speed and accuracy of the information. I work at a company that uses AI services, and AI helps to build the foundation of the work, after which the content is checked and refined by a human.
The ability to research across various sources, creating more complete and informative responses.
Access to information in an easy and practical way.
More information
Immediate responses.
Ease of interaction with the software using natural language.
I think it's a positive factor, the ability to search a large database.
Simplified language.
Accessible language, well-founded sources.
Synthesis ability, clear and accessible language.

Question 3

Based on the experience gathered in evaluating the generated answers, in what ways do you think AI models could improve in their role as a source of high-quality medical information?

Answer

Not sure if it is possible for AI to incorporate patient's cultural backgrounds or level of health literacy before generating answers to patient questions. If this would be possible, it would be powerful and helpful.
Including benefit/risk information. Including reliable sources for more information.
Providing sufficient information to make informed choices.
A useful supplement to the multitude of questions asked of OBs during a short clinic visit and to support our triage RNs.
There are many common questions that everyone asks. Responses to common questions could be helpful
NA
Incomplete in some responses,
I think it would be wonderful way to provide answers to patients who are asking questions through patient portals.
Yes
As a tool for the patient to clarify questions they have in their daily life.
NA
As a means of generating educational texts to guide patients with more accessible language.
NA
No doubt

More personalized and clear.
NA
NA
simple language
By using brief and simple knowledge
NA
NA

Cannot be too medicalized and need to acknowledge individuals who vary from recommendations. Maybe give references for more information and should always have reasons to contact provider.
if curated for accuracy.
NA
NA
Confirming accurate and complete information. Providing additional resources. Unsing empathetic language.
Access is still limited to an audience with broad access to the internet and language models. There is a lack of studies evaluating the usefulness, safety, and quality of the outputs of LLMs for the general public.
Specify that the procedures and treatments depend on each case and should always be evaluated by the doctor.
Providing more complete answers, sourced from reliable references.
I believe that the AI's source of information is crucial in shaping the responses.
I think the models are good, but they need to be thorough and based on the best scientific evidence.
Detailed explanations
evidence based advice in simple language
NA
NA
By doing better Urdu translation
Yes
Providing the main sources at the end and showing the different possibilities (without generalizing into just one) within a topic.
Being based on current evidence.
NA
Information based on literature data.
There is no mention of the information source, making it difficult to ensure the accuracy of the information.
Provide some sources that the reader can consult if they want to seek more information. Additionally, make it clear (in questions related to medicine) the importance of medical follow-up.
Use a scientific database.
Always use data from public institutions such as the Ministry of Health (MS), WHO (World Health Organization).
Source of scientific and high-quality research.

Intelsurv — Question 1

Do you think IntelSurv has the potential to improve your access to knowledge in your work?

Answer

Yes
Yes
Yes
Yes
Yes
Yes
Yes

Yes
Yes
Yes
Yes
Yes
Yes
Yes
Yes
Yes
Yes
Yes

Question 2

Participant Impression about Intelsurv

Answer

Intelsurv has come in a right time and I believe it has eased the work that we had before, for instance, at first, we could find it more difficult to rule out whether it was indeed the disease in question or not but this tool has helped us to define the disease/condition with much ease. This intelsurv will also help in having a complete line listing since it captures every important aspect unlike in the past where one could miss out some information of a client.

The intelsurv is very important because the tool that has been developed will help us to give direction on some data elements for example, the importance of taking the names of the guardian when filling the form. We will be able to explain better to the clients as this helps us during the follow-up visit

This app is very useful in terms of providing real-time support on case definitions and information about the fields and/ the form. In disease surveillance, data quality is key which begins with collecting the right data, by understanding what is required for each field on the form. Intelsurv would be very useful for novel diseases and conditions for workers to have real-time answers for case definitions etc. For example, if we are to have Ebola, correctly classify one as having a disease or not, case definitions would be easily generated by the app. Secondly, since it's quite challenging to orient everyone on how to fill a case-based surveillance, the app has capacity to aid one to fill the form without any challenges. Overall, the great anticipated challenge would be internet access in some areas that do not have network coverage. Recommend having an offline option for pre-defined diseases and conditions, case definitions and also definitions for the fields on the form.

The intelsurv could have been more useful if it was developed a long time ago when covid-19 became a burden to almost the whole world because it was difficult to answer some of the questions which the application is answering. It is still more useful to disease surveillance because we are still experiencing some epidemics which need surveillance like the cholera burden which is being experienced right now in Malawi.

The Intelsurv app is very easy to use and understand and with this technology work will be easy when dealing with surveillance diseases. Back then when covid-19 was at its highest peak, it was very difficult for us because we did not have much knowledge because the disease was new but now with this app it will be easy to know some of the information by just asking the tool questions.

Data can easily be kept for future use, user friendly

The tool is very helpful in times when for example the patient has no guardian and is unconscious, it can help to dig more about the questions if the name and other information is less. When you have received a case based form which is under filled, the tool can help in identifying the missing information and dig more about it.

It has guiding questions and it's easy to access the information of different diseases and can also be used in outpatient departments

It is good and more forms to the area of study or the case at hand. Therefore this makes it as a priority of forms for use

Intelsurv is going to help the one filing case based form in a way that there will be no missed information because he/she is not understanding, this will help to guide the person. It gives direct information for easy use and the information is very understandable to the person using it.

The Intelsurv is the way to go as it has almost every answer right there, it is very easy to use because I personally managed to do everything on my own without seeking any help from the facilitators. However, my suggestion is that, would you please if possible make it to produce words as someone navigates, there are some people who have eye problems but are able to hear properly. Make it to produce words.

Intelsurv serves a purpose to new reporters to understand and fill the case base forms with ease. It also helps to standardize reports with universal description. But, it might not serve a purpose to fill the gaps in data collection because it does not provide the needed missing information but rather explains what is needed. Perhaps educating the reporters in the health centers would cater for the other purpose of providing all details.

It is a very nice application. Well informed and easy to use in times of emergencies. There were some internet issues only when using the application while some apps were working effectively. Regardless, it's well designed and easy to understand even for a non-medical person. Likely to use the application often.

It is providing enough information per each data element you ask a patient or additional information per each disease.
Basically it is useful because it is able to answer all the questions in case someone has forgotten all he/she needs a clarification in a certain area or question. It is a nice development. I am likely to use it when I need it.
Its user friendly and can be useful in times of public health emergencies
Intelsurv is very useful because it is able to answer all the questions in case someone has forgotten all he/she needs a clarification in a certain area or question. It is a nice development. I am likely to use it when I need it.
The developed tool is easy to use. You can use your data if you are asking your own question but if you ask an already made question no data is used and you can easily fill the case based form, if you find/meet a difficult question you go back to the tool which is simple. Case management is simple using the tool.

Question 3

General Comments on the tool

Answer

The app is generally easy to use, and very fast although when you ask it alot of information at once it takes time. The app should be used for different programs. It is very useful
The tool is very useful because it can be used to search for other general conditions. It will reduce time used in searching information
The tool is well designed, the tool is user friendly, the tool will be useful in sourcing some medical information and challenges we encounter especially on that page of frequently asked questions
The tool is very useful because sometimes you may need referrence of other diseases and it help alot. But it needs to be upload on tablets also for those with tablet
Very important tool and helpful in answering medical general information
The tool is well formulated, it is able to answer critical questions
It is a good tool, it will help in correctly filling of the case based forms because it is acting as a referrence for correctly filling the form and understanding the variables indicated on the form
The tool is very useful as you can be assisted in any field which means can be used anytime when needed. The tool can be used when offline
The tool is so important and is also helpful and also its easy to use it
The tool is very important in a way that it's easy to understand it and it gives information
The tool is very useful, it will be useful on our work
Well, the tool is very interesting and easy to use and has got all the necessary infomation concerning health related issues
I was getting all answers correctly and timely and in a well detailed manner. The app will help and simplify the work and it will help because data is going to be kept safe all the time as compared to paper based kind of keeping data
The app seems to be fast, good and friendly. Try to add more features on them and try to be friendly on mobile small phones
The tool is fine to work with it but some phones are not working with that tool
This app can work even in other fields, very important and useful, makes the user free from responses during and after school
This has come at the right time and its good
Very easy to use, very useful to work

Question 4

Any issues that the paticipant observed when interacting with Intelsurv

Answer

No major issues apart from taking time when you have requested a lot of information at once
Easy to use, The tool will stop people use their brains (not thinking)
The tool has no issues and can be used at all levels
Nice and needs to be roll out so that many people can start using it. Improve so that all kinds of phones should use the app
It was nice to use and user friendly
Easy to use, Well detailed
It is giving unlimited information that is asked, it is easy to use
The tool is very good and user friendly
This app is also good interms of asking questions it's easy to get answers from this app
The intersurv make life easy because it is skilled and gives or answers any question in an easy way
Very fast and reliable and can have any information from this app concerning case definition

		Somehow the app could not be able to disminate other vital information
		The app is friendly and simple to use. The problem is our gadgets that have not enough space but I think most people can easily use it<br>The tool is fine as it gives us information what ever question we asked<br>No any issue, I just feel the application at the same time I like it. I would like during launch, it should be done here in Blantyre. If its possible add Chichewa version in it.<br>What I observed is that some of the things on case definitions are not yet on the tools but I hope they will be added<br>It is providing correct information sought
HA Clinics	Question 1	Feedback on how useful was the utilization of the LLM
	Answer	Good<br>By providing me with useful and credible suggestions.<br>By giving appropriate suggestions and feedback<br>By providing the appropriate suggestions<br>In terms of giving the suggestion for the test to carry out and the medication<br>By giving the appropriate suggestions<br>By providing the appropriate suggestions<br>I have been improved by the decision of the LLM to do some investigation such as neurological assessment of the patient.<br>Improved by suggesting some test and the diagnosis.<br>My patient care<br>It's remind me the patient condition and help in treatment plan<br>Improved my treatment plan<br>It's improved my treatment plan<br>Improved my treatment plan<br>Improved my treatment Plan<br>Improved my treatment plan<br>Improved my treatment plan<br>Improved my treatment plan<br>Improved my treatment plan<br>My care has been improved by the LLM suggestions.<br>Improved my knowledge of diagnosis<br>Improved my treatment plan<br>Improved my treatment plan<br>Improved my treatment plan<br>By providing the appropriate suggestions for the client<br>By providing appropriate suggestions<br>By providing appropriate suggestions for the patient.<br>By providing appropriate suggestions for the patient<br>By providing appropriate suggestions for the patient<br>By providing the appropriate suggestions for the patient.<br>Improved my treatment plan<br>Through given suggestions on patient care and management<br>In term of management of patients and the mode of diagnosis and assessment<br>In term of diagnosis and management<br>In term of patients care and management<br>Regarding the management of patients condition

	By making suggestions that i did not remember.
	Has improved my care by helping me to make a proper diagnosis
	Help me provide better care for the patient
	By providing the appropriate suggestions for the patient.
	By providing the appropriate suggestions for the patient
	By providing appropriate suggestions for the patient
	By providing the appropriate suggestions for the patient
	By providing the appropriate suggestions for the patient.
	By providing the appropriate suggestions for the patient
	In term of diagnosis and management
	In term of diagnosis and treatment of patients
	In term of patients care and management
	By providing the appropriate suggestion for the patient.
	It highlights me on what am supposed to add or adjust in my diagnosis.
	Improved my treatment plan
	Good
	Good
